# The effect of enhanced recovery after surgery in laparoscopic sacrocolpopexy: a randomized controlled trial

**DOI:** 10.1097/JS9.0000000000004621

**Published:** 2026-02-12

**Authors:** Sisi Deng, Qianchun Gu, Limin Liu, Xiaofeng Lei, Jin Yu, Xuezhu Huang

**Affiliations:** a Department of Anaesthesiology, Chongqing Health Center for Women and Children, Chongqing, China; b Department of Anaesthesiology, Women and Children’s Hospital of Chongqing Medical University, Chongqing, China; c Department of Anaesthesiology, Qijiang Health Center for Women and Children, Chongqing, China

**Keywords:** enhanced recovery after surgery, hospital costs, laparoscopy, length of stay, pelvic organ prolapse

## Abstract

**Background:**

The enhanced recovery after surgery (ERAS) protocol has been demonstrated to improve patient outcomes; however, its efficacy has not been prospectively evaluated in randomized controlled trials (RCTs) for patients undergoing laparoscopic sacrocolpopexy (LSC). This study aimed to develop a multidisciplinary ERAS protocol specifically for LSC and to systematically evaluate its clinical efficacy and benefits following implementation.

**Materials and Methods:**

In this prospective RCT conducted between September 2023 and February 2024, 80 patients undergoing LSC were randomized to either ERAS protocol (Group E) or conventional perioperative care (Group C). Primary outcomes included postoperative length of stay (LOS) and hospitalization costs, with secondary outcomes assessing recovery parameters, complication rates, pain scores, vital signs, and laboratory markers.

**Results:**

Implementation of the ERAS protocol significantly reduced median postoperative LOS [77.00 (69.00–87.00) h vs. 46.50 (40.00–50.00) h; median difference: 31.00 h, 95% CI: 26.00–36.00; *P* < 0.001]. The ERAS group showed statistically significant improvements in multiple recovery metrics: earlier urinary catheter removal (median reduction 12 hours), quicker resumption of oral intake (18 hours), faster return of bowel function (8 hours), and earlier ambulation (4 hours) (all *P* < 0.001). Group E exhibited significantly improved 7-day QoR-15 scores (*P* = 0.002), reduced pain scores, decreased opioid use, lower postoperative white blood cell counts, and fewer episodes of postoperative nausea and vomiting. The 30-day readmission and emergency department visit rates in both group were zero.

**Conclusion:**

This RCT establishes that implementation of an ERAS protocol for LSC significantly enhances postoperative recovery, reduces pain and complications, decreases opioid use while attenuating surgical stress responses. The 40.26% reduction in (LOS) meets the minimum clinically important difference threshold, indicating a clinically meaningful improvement. Additionally, the 30-day readmission and ED visit rates remained zero, further confirming the safety of the ERAS protocol in low-risk women.

## Introduction

Enhanced recovery after surgery (ERAS) is a multidisciplinary care approach that incorporates evidence-based interventions aimed at optimizing the surgical care process from treatment initiation to recovery^[1]^. The first ERAS pathway was developed in Europe for colorectal surgery and has since been adapted for other surgical specialties, including orthopedic, urologic, thoracic, and obstetric surgery^[2–4]^. After ERAS implementation, patients experienced a decreased length of stay (LOS), hastened return of bowel function, improved postoperative pain control, and high patient satisfaction^[5,6]^.

HIGHLIGHTS

- A single-center, prospective, randomized controlled trial was conducted on the clinical safety and effectiveness of peri-operative ERAS protocol in laparoscopic sacrocolpopexy.
- Patients undergoing laparoscopic sacrocolpopexy were randomly assigned to Group E (ERAS protocol) or Group C (conventional care)
- ERAS protocol significantly reduces postoperative length of stay (46.5 h vs. 77.0 h).
- ERAS protocol expedited urinary catheter removal, oral intake, ambulation, and intestinal recovery.
- Patients in Group E reported improved 7-day postoperative QoR-15 scores.
- ERAS protocol reduced postoperative pain, opioid use, complications, and inflammation.

Laparoscopic sacrocolpopexy (LSC) is a minimally invasive treatment for pelvic organ prolapse (POP), a common condition in women that significantly affects their quality of life^[7]^. As life expectancy increases, the global need for cost-effective care for women with POP is expected to increase^[8]^. However, current research on ERAS protocols specifically for LSC is limited, with most studies being observational or retrospective in nature^[9,10]^, and there is a notable lack of high-quality prospective randomized controlled trials (RCTs) to support these findings^[11]^. Whether certain ERAS strategies that have shown positive effects in other types of surgery can be safely applied to LSC remains unclear. For example, in early postoperative removal of urinary catheters^[1,3]^, the incidence of postoperative urinary retention (POUR) is relatively high after pelvic floor reconstructive surgery, often necessitating prolonged indwelling catheterization^[12]^, and early catheter removal may increase the risk of POUR in patients with LSC. In addition, the optimal combination and dosage of multimodal analgesia techniques and medications for patients with LSC, the potential impact of shortened LOS on readmission rates, and the adherence of both healthcare providers and patients to ERAS protocols^[13]^, are all pressing issues that need to be addressed.

Given these knowledge gaps, we designed and conducted a prospective RCT to evaluate the efficacy of the ERAS protocol compared to conventional treatment in patients undergoing LSC. We hypothesized that implementing an ERAS protocol in these patients will enhance postoperative recovery, including reductions in LOS, hospital costs, and other key outcomes, compared to conventional perioperative care.

## Materials and methods

### Study design and ethical regulations

This was a single-center, prospective RCT of patients who underwent elective LSC between September 2023 and June 2024 at the Women and Children’s Hospital of Chognqing Medical University. The study received ethical approval from the Medical Ethics Committee of Women and Children’s Hospital of Chognqing Medical University (Approval number: 2023-039) and was retrospectively registered with the Chinese Clinical Trial Registry (Registration number: ChiCTR2400087431, the URL of registration is https://www.chictr.org.cn/showproj.html?proj=228919). All participants provided written informed consent, and the protocols strictly adhered to the principles of the Declaration of Helsinki. The article is compliant with the TITAN Guidelines 2025^[14]^. The work has been reported in line with Consolidated Standards of Reporting Trials (CONSORT) Guidelines^[15]^.

### Sample size calculation

The sample size was calculated using the Power Analysis and Sample Size (PASS) software, version 2024 (NCSS, LLC, Kaysville, USA). Currently, the average postoperative LOS for patients undergoing LSC is 79.00 ± 19.27 hours in our medical institution. We found that implementing an ERAS protocol could reduce postoperative LOS by 15 hours compared to the conventional care group in pilot study. We used two-sample *t*-tests, assuming equal variance, to calculate the sample size, setting a significance level (alpha) of 0.05 and targeting a power of 90%. The results indicated that each group required a sample size of 36. After factoring in a 10% dropout rate, this required a minimum of 40 patients per group, for a total of at least 80 participants. Postoperative LOS was considered the first primary endpoint and hospitalization cost was the second primary endpoint. No multiplicity adjustment was applied; if Bonferroni-corrected (α = 0.025), the conclusion remained unchanged.

### Inclusion and exclusion criteria

All patients were evaluated for eligibility before hospital admission. The inclusion criteria were patients aged 18–60 years, American Society of Anaesthesiologists (ASA) physical status I or II, scheduled for elective LSC, and signed the informed consent. The exclusion criteria were complications such as gynaecological malignant tumours, acute infections, diabetes, mental illnesses, and other contraindications for surgery, including those related to use of nonsteroidal anti-inflammatory drugs (NSAIDs) such as peptic ulcers, known NSAIDs allergies, and a history of aspirin-induced asthma. Patients who required emergency surgery were excluded. Withdrawal criteria included patients refusal to continue the study protocol or the need for an unexpected extension of surgery.

### Study outcomes

The primary outcomes were postoperative LOS and total hospitalization costs. Postoperative LOS was defined as the hours from the completion of surgery to readiness for hospital discharge. Secondary outcomes were the other rehabilitation indices [time to first oral intake, first ambulation, first intestinal exhaust, time to urinary catheter removal, quality of recovery scores, patient satisfaction evaluation scores and post-void residual urine volume (PVR)]; postoperative complications, postoperative pain scores, vital signs, and routine blood tests. The quality of recovery scores was evaluated using the QoR-15, which consists of 15 items related to clinical recovery, including pain, sleep quality, emotional state, nausea and vomiting, and overall health. Each item is scored on a scale of 1 to 10, with a maximum possible score of 150. A higher score indicates a better quality of postoperative recovery^[16]^. Postoperative pain was evaluated using a numeric rating scale (NRS) ranging from 0 to 10, where 0 indicates no pain, and 10 indicates the worst possible pain^[17]^. Patient satisfaction evaluation scores ranged from 0–10, where 0 indicates extreme dissatisfaction, and 10 represents extreme satisfaction.

Postoperative complications included pruritus; injury of urinary system or intestinal canal; POUR, characterized by PVR exceeding 100 mL; intestinal obstruction, confirmed through clinical symptoms and imaging; postoperative respiratory tract infections, confirmed through clinical symptoms and positive pathogen culture; incision infections, evidenced by positive pathogen culture; postoperative deep venous thrombosis, verified via symptoms and ultrasound; readmission or emergency department (ED) visit within 30 d postoperatively due to factors related to the surgery or treatment; and postoperative nausea and vomiting (PONV) grading (Grade I—no nausea; Grade II—mild nausea, mild abdominal discomfort, no vomiting; Grade III—evident nausea and vomiting, but no material expelled; Grade IV—severe vomiting, expulsion of gastric contents necessitating medication).

Episodes of vital sign instability included hypotension/hypertension, defined as a decrease or increase in mean blood pressure (MBP) > 30% compared to pre-anesthesia levels; bradycardia/tachycardia, with a heart rate (HR) < 50 bpm or > 100 bpm; and hypoxaemia, indicated by oxygen saturation <92% despite supplemental oxygen.

### Randomiation, allocation, and concealment

Prior to admission, patients were randomized in a 1:1 ratio to either the conventional care group (Group C) or the ERAS group (Group E) using a computer-generated random number list. Group C received the conventional perioperative care protocol, whereas Group E followed the ERAS protocol. Due to the need for clinical staff to conduct patient education, prescribe medications, and perform nerve blocks, complete blinding was not feasible. However, to mitigate bias in the reporting of subjective outcomes (e.g., QoR-15, pain scores), data collection and outcome assessments were performed by blinded assessors who were independent from the clinical care teams and were unaware of the group assignments throughout the study. These blinded assessors had no access to patient records or group allocation.

### Study protocol

**Figure 1.**
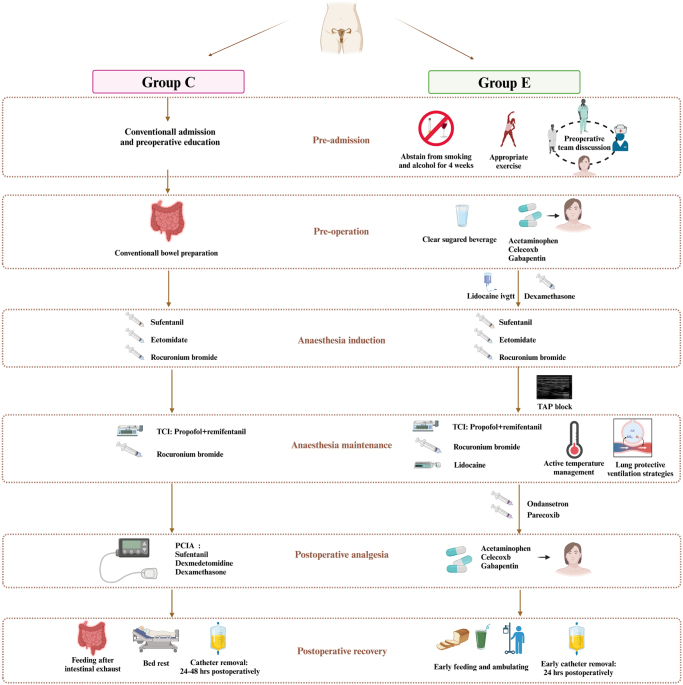
Graphical abstract.

After conducting a literature review of the established ERAS protocols and considering the specific conditions of patients with POP and the characteristics of the surgery, we developed a multidisciplinary ERAS protocol. A more detailed comparison of the conventional care and ERAS protocols for LSC is shown in Figure 1. All enrolled patients underwent elective LSC performed by the same experienced surgical team and received the assigned interventions.

PCIA = patient-controlled intravenous analgesia; TCI = target-controlled infusion.

#### Preoperative elements

In Group C, patients were admitted according to the conventional procedures and underwent bowel preparation the night before surgery by taking an oral laxative. They were instructed to observe an 8-h fasting period for solid foods and a 2-h restriction on clear liquid intake.

In Group E, patients ceased smoking and alcohol consumption 4 weeks prior to surgery and engaged in regular exercise daily. Any existing anemia was treated. Upon admission, a collaborative effort involving surgeons, anesthesiologists, and nurses was undertaken to educate patients on the objectives of ERAS, perioperative management processes (including surgical and anesthetic procedures), steps the patients should comply with, and postoperative recovery. Patients were instructed to observe an 6-h fasting period for solid foods. Two hours before surgery, patients consumed a 300 mL of a clear sugared beverage (each 100 mL contained 12.5 g carbohydrates, 50 mg sodium, 66.7 mg potassium, 63 mg phosphorus, and 65 mg chloride) and were administered NSAIDs for pain management, including gabapentin 300 mg, paracetamol 500 mg, and celecoxib 200 mg.

#### Intraoperative elements

In Group C, the anesthesia induction sequence consisted of sufentanil at a dose of 0.4–0.5 μg/kg, etomidate at 0.4–0.5 mg/kg, and rocuronium at 0.6 mg/kg. After induction, an endotracheal tube was inserted and connected to the anesthesia machine in volume-controlled ventilation (VCV) mode to maintain the end-tidal carbon dioxide pressure within the range of 35–40 mmHg. Anesthesia was maintained through target-controlled infusion (TCI) of propofol and remifentanil, with additional rocuronium bromide administered at 0.15 mg/kg every 30 min. Intraoperative temperature was monitored, and warming devices were used if the temperature dropped below 36°C. All patients received Ringer’s solution (Na^+^ 147 mmol/L, K^+^ 4 mmol/L, Ca^2+^ 4 mmol/L) as the primary intraoperative fluid. Maintenance rates were estimated using the “4–2—1” rule^[18]^ and adjusted for one-half to two-third of the preoperative fasting deficit, third-space losses (1–2 mL/kg/h), and blood loss.

In Group E, an intravenous infusion of lidocaine (1 mg/kg) and an injection of dexamethasone (5 mg) were administered before anesthesia induction, following the same protocol as in Group C. After endotracheal intubation, a transversus abdominis plane (TAP) block was performed under ultrasound, with an injection of 15 mL of 0.5% ropivacaine at each block site. Anesthesia maintenance included TCI of propofol, remifentanil, and intermittent boluses of rocuronium bromide, supplemented with a continuous lidocaine infusion at 1 mg/kg/h. At the end of surgery, patients received 40 mg of parecoxib and 5 mg of ondansetron, or another 10 mg of metoclopramide when PONV risk score was ≥3^[19]^. Active temperature management was used intraoperatively to maintain body temperature between 36°C and 37°C. In addition, a lung-protective ventilation strategy was employed, which included low tidal volume (6–8 mL/kg), low positive end-expiratory pressure (PEEP) ventilation (5–8 cmH^2^O), and lung recruitment were performed every 30–60 minutes (pressure-controlled ventilation (PCV) at 20 cmH^2^O with PEEP of 10 cmH^2^O, sustained for 10 breaths). Due to ingestion of a sugared beverage 2 hours before surgery, preoperative fluid deficit was reduced by approximately half, and intraoperative fluid therapy was adjusted accordingly.

Throughout the surgical procedures, both groups maintained a bispectral index within the range of 40–60. In instances where the HR fell below 50 bpm, atropine at a dose of 0.3 mg was administered; conversely, if the HR exceeded 100 bpm, the anesthesia depth was increased. MBP was meticulously managed to fluctuate within 30% of the preoperative baseline value; should there be a decline surpassing 30% of the baseline range, ephedrine was administered at a dosage of 3–5 mg; if the increase exceeded 30% of the baseline range, the anesthesia depth was augmented accordingly.

#### Postoperative elements

In Group C, postoperative pain management was provided through a patient-controlled intravenous analgesia (PCIA) pump containing 100 μg of sufentanil, 100 μg of dexmedetomidine, and 10 mg of dexamethasone, diluted in 0.9% saline solution to a total volume of 100 mL. The infusion was set at a continuous rate of 2 mL/h with a bolus dose of 2 mL and a lockout time of 15 min. Postoperatively, the patients adhered to the traditional practice of bed rest and began feeding only after the intestinal exhaust. The urinary catheter was removed 24–48 hours after surgery.

In Group E, patients received oral analgesics instead of PCIA. Upon regaining consciousness, they began oral intake of water and analgesics, including gabapentin 300 mg, paracetamol 500 mg, and celecoxib 200 mg, every 8 hours. Early postoperative feeding was initiated 3 h after surgery, and the urinary catheter was removed 24 h postoperatively. The patients were encouraged to set daily activity goals and ambulate as early as possible during the postoperative period.

All patients were scheduled for structured outpatient follow-up at 7 and 30 days after surgery. During these visits, post-discharge complications, ED visits, and any other unplanned healthcare utilization were recorded. The QoR-15 questionnaire was also administered at both follow-up points to assess recovery quality.

### Discharge criteria

Patients were discharged when they met all the following discharge criteria: tolerating a soft/regular diet, no longer requiring intravenous antibiotic therapy, adequate pain control (NRS scores <4); good wound healing well with no signs of infection, good general condition with the ability to ambulate freely, and without urinary catheters.

### Statistical analysis

All statistical analyses were performed using Statistical Package for Social Science (SPSS) for Windows, version 21.0 (SPSS Inc., Chicago, IL, USA). Normality was assessed using the Shapiro–Wilk test. The quantitative data, which exhibited a normal distribution, were presented as mean ± standard deviation and compared between the two groups using the Student’s *t*-test, with effect sizes expressed as mean differences and 95% confidence intervals (CI). In contrast, the quantitative data with an abnormal distribution were presented as median (interquartile range) and compared using the Mann–Whitney U-test, with effect sizes expressed as Hodges–Lehmann estimates (median differences) and corresponding 95% CI. The generalized estimation equation was used to test repeated measures, such as vital signs, NRS scores for pain, white blood cell (WBC) count, and hemoglobin (HGB). Categorical data are presented as numbers (percentages) and were compared using the Pearson’s chi-squared and Fisher’s exact tests. The Mann–Whitney U-test was used to compare satisfaction evaluation scores and PONV. Comparisons for the primary outcomes were performed using general linear models adjusted for baseline variables with standardized mean differences (SMD) > 0.1. Statistical significance was set at a two-tailed *P*-value < 0.05. Secondary analyses were exploratory and not adjusted for multiplicity; findings require confirmation in future RCTs.

## Results

**Figure 2.**
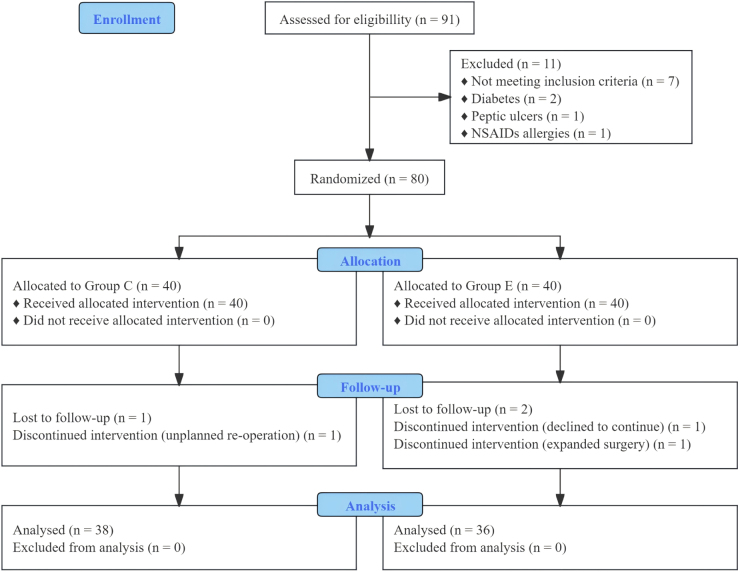
Flow chart of the study.

During the study period, from September 2023 to June 2024, 80 patients were screened for eligibility and subsequently randomized into two groups. Three patients were lost to follow-up, one patient withdrew due to refusal to continue the study protocol, and two patients were excluded from the analysis due to unplanned surgeries (Fig. 2).

### Patient demographic characteristics

The baseline demographic characteristics, including age, weight, height, and ASA physical status classification were in Table 1.

**Table 1:** Characteristics of patients receiving laparoscopic sacrocolpopexy.

Characteristic	Group C (*n* = 38)	Group E (*n* = 36)	Standardized mean differences (SMDs)
Age, years	53.00 (49.00, 57.00)	50.00 (39.00, 58.00)	0.242
ASA (I/II), *n*	24/14	26/10	0.195
Height, cm	155.00 (152.00, 160.00)	158.00 (155.00, 160.00)	0.409
Weight, kg	57.00 (55.00, 62.00)	55.00 (50.00, 65.00)	0.309
Physical condition (hypertension/arrhythmias/cardiovascular disease), *n*	8/2/0	6/2/2	0.343
Smoking status, *n* (%)	4 (10.53)	0	0.485
Alcohol use, *n* (%)	0	0	0

Data are given as median (inter-quartile range) (Shapiro–Wilk *P*-values < 0.05) and *n* (%).

ASA = American Society of Anesthesiologists.

### Primary outcomes

The postoperative LOS in Group E was significantly shorter than that in Group C (77 (69.00, 87.00) h vs. 46.5 (40.00, 50.00) h, median difference: 31.00 h, 95% CI: 26.00–36.00, *P* < 0.001). A sensitivity analysis using a worst-case scenario was performed to assess the impact of missing data. All randomized patients (*n* = 80) were included, with the two missing patients from the ERAS group assigned an extreme LOS value of 200 h. The beneficial effect of the ERAS protocol on reducing LOS remained statistically significant (*P* < 0.001). whereas there was no

significant difference in hospitalization costs between the two groups (Table 2).

**Table 2:** Postoperative recovery data.

Characteristic	Group C (*n* = 38)	Group E (*n* = 36)	Median difference (95% CI)	*P-*value
Primary outcomes				
Postoperative LOS, h	77.00 (69.00, 87.00)	46.50 (40.00, 50.00)	31.00 (26.00, 36.00)	<0.001*
Hospitalization Costs, RMB	19 807.00 (17 847.00, 21 565.00)	19 117.00 (18 094.00, 20 798.00)	980.50 (−223.00, 1838.00)	0.056
Other rehabilitation indexes				
Time to first oral intake, h	20.00 (19.00, 25.00)	3.00 (2.00, 3.50)	18.00 (17.00, 19.00)	<0.001*
Time to first ambulation, h	22.00 (17.00, 24.00)	18.00 (12.00, 21.00)	4.00 (2.00, 7.00)	<0.001*
Time to first intestinal exhaust, h	19.00 (14.00, 24.00)	12.00 (7.00, 16.50)	8.00 (6.00, 12.00)	<0.001*
Time to urinary catheter removal, h	36.00 (28.00, 40.00)	23.50 (20.00, 26.00)	12.00 (8.00, 15.00)	< 0.001*
7-Day postoperative QoR-15 scores	146.00 (142.00, 147.00)	147.50 (146.00, 149.00)	−2.00 (−1.00, − 3.00)	0.004*
30-Day postoperative QoR-15 scores	150.00 (150.00, 150.00)	150.00 (150.00, 150.00)	0.00 (0.00, 0.00)	1.000
Patient satisfaction evaluation scores	9.50 (8.00, 10.00)	10.00 (9.00, 10.00)	0.00 (−1.00, 0.00)	0.061
Post-void residual urine volume (PVR), mL	15.50 (11.00, 22.00)	14.5 (10.25, 21.00)	1.00 (−4.00, 5.00)	0.696

Data are given as median (inter-quartile range) (Shapiro–Wilk *P*-values < 0.05).

^*^ *P* < 0.01, likely true positive; ^**^ 0.01 ≤ *P* ≤ 0.05, hypothesis-generating.

CI = confidence interval; LOS = length of stay; PVR = post-void residual urine volume.

### Secondary outcomes

#### Other rehabilitation indexes

Patients in Group E had a significantly shorter time to first feeding after surgery, compared to Group C (20.00 (19.00, 25.00) h vs. 3.00 (2.00, 3.50) h, median difference: 18.00 h, 95% CI: 17.00–19.00, *P* < 0.001). The postoperative time for urinary catheter removal was also significantly shorter in Group E than in Group C (36 (28.00, 40.00) h vs. 23.5 (20.00, 26.00) h, median difference: 12.00 h, 95% CI: 8.00–15.00, *P* < 0.001). Similarly, the time to first intestinal exhaust and ambulation were shorter in Group E. The QoR-15 scores on postoperative day 7 were significantly higher in Group E than in group C. There was no statistically significant difference in the patient satisfaction evaluation scores and PVR between the two groups(Table 2).

#### Surgery and anesthesia data

There were no significant differences between the two groups in terms of operative time, urine output, blood loss, extubation time, or vasopressor use. However, the intraoperative fluid intake and consumption of propofol and remifentanil were significantly lower in Group E (Table 3).

**Table 3:** Surgery and anesthesia data.

Characteristic	Group C (*n* = 38)	Group E (*n* = 36)	*P-*value
Operative time, min	140.00 (105.00, 175.00)	113.50 (97.00, 165.00)	0.109
Infusion volume, mL	900.00 (800.00, 1000.00)	750.00 (500.00, 900.00)	0.003*
Urine volume, mL	120.00 (100.00, 200.00)	150.00 (100.00, 200.00)	0.821
Bleeding volume, mL	30.00 (30.00, 50.00)	40.00 (30.00, 50.00)	0.256
Extubation time, min	8.00 (6.00, 10.00)	7.50 (6.00, 9.00)	0.457
Propofol consumption, mg	1110.00 (920.00, 1320.00)	820.00 (660.00, 1120.00)	< 0.001*
Remifentanil consumption, μg	1300.00 (920.00, 1600.00)	690.00 (600.00, 1000.00)	< 0.001*
Ephedrine consumption, mg	0.00 (0.00, 6.00)	0.00 (0.00, 6.00)	0.443
Atropine consumption, mg	0.00 (0.00, 0.00)	0.00 (0.00, 0.00)	0.388

Data are given as median (inter-quartile range) (Shapiro–Wilk *P*-values < 0.05).

^*^ *P* < 0.01, likely true positive; ^**^ 0.01 ≤ *P* ≤ 0.05, hypothesis-generating.

#### Postoperative complications

No serious postoperative complications occurred in either group, including urinary retention, ileus, infection, or deep venous thrombosis. No patient in either group experienced unplanned readmission, ED visits, or other unplanned healthcare utilization within 30 days after surgery. While there was no significant difference in the incidence of PONV within the first 24 h postoperatively, Group E exhibited a lower incidence of PONV at 48 h postoperatively (Table 4).

**Table 4:** Postoperative complications.

Characteristic	Group C (*n* = 38)	Group E (*n* = 36)	*P-*value
Pruritus, *n* (%)	0	0	/
Intestinal obstruction, *n* (%)	0	0	/
Respiratory tract infection, *n* (%)	0	0	/
Incision infection, *n* (%)	0	0	/
Deep venous thrombosis, *n* (%)	0	0	/
Injury of urinary system or intestinal canal, *n* (%)	0	0	/
Urinary retention, *n* (%)	0	0	/
Readmission or emergency department visits within 30 days postoperatively, *n* (%)	0	0	/
2 h PONV(I/II/III/IV), *n*	28/6/4/0	30/4/0/2	0.335
12 h PONV(I/II/III/IV), *n*	28/6/2/2	30/4/0/2	0.320
24 h PONV(I/II/III/IV), *n*	30/8/0/0	30/6/0/0	0.632
48 h PONV(I/II/III/IV), *n*	34/4/0/0	36/0/0/0	0.047**

Data are given as *n* (%).

^*^ *P* < 0.01, likely true positive; ^**^ 0.01 ≤ *P* ≤ 0.05, hypothesis-generating.

PONV = postoperative nausea and vomiting.

PONV graded criteria in four grades: Grade I – no nausea, Grade II – mild nausea, mild abdominal discomfort, no vomiting, Grade III – evident nausea and vomiting, but no material expelled, Grade IV – severe vomiting, expulsion of gastric contents necessitating medication.

#### Perioperative pain

**Figure 3.**
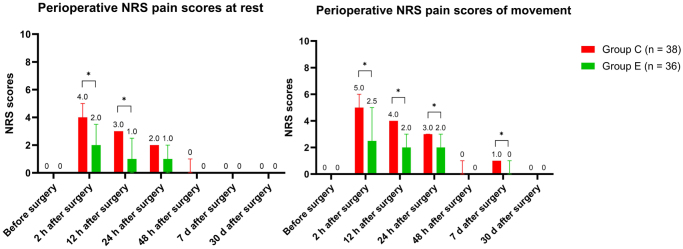
Perioperative NRS pain scores.

In the first 12 h postoperatively, patients in Group E had significantly lower NRS pain scores at rest and during movement than those in Group C. Additionally, the relief of movement-related pain in Group E persisted up to 24 h postoperatively, with this difference remaining statistically significant even 7 days after surgery (Fig. 3). Moreover, within the first 12 h postoperatively, significantly fewer patients in Group E required additional analgesia for pain scores exceeding 3 than those on Group C [20 (52.6) vs. 10 (27.8), *P* = 0.030] (Table 5).

* Significant differences between two groups (*P* < 0.05).

Abbreviations: NRS = numerical rating scale.

0 denoted “no pain” and 10 denoted “worst pain imaginable.”

**Table 5:** Postoperative additional analgesia.

Characteristic	Group C (*n* = 38)	Group E (*n* = 36)	*P-*value
2 h postoperative additional analgesia, *n* (%)	20 (52.63)	10 (27.78)	0.030**
12 h postoperative additional analgesia, *n* (%)	10 (26.32)	2 (5.56)	0.015**
24 h postoperative additional analgesia, *n* (%)	2 (5.26)	0	0.494
48 h postoperative additional analgesia, *n* (%)	0	0	/

Data are given as *n* (%).

^*^ *P* < 0.01, likely true positive; ^**^ 0.01 ≤ *P* ≤ 0.05, hypothesis-generating.

#### Vital signs and blood routine tests

**Figure 4.**
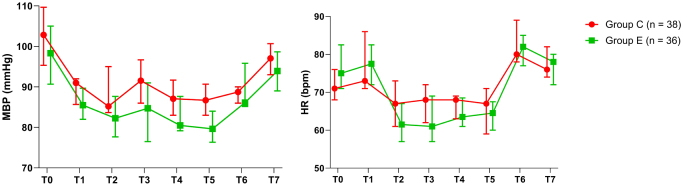
Changes in hemodynamic parameters.

There were no statistically significant differences between the two groups in perioperative MBP and HR at any of the measured time points (Fig. 4). Additionally, the incidence of perioperative episodes of vital sign instability was similar between groups (Table 6).

* Significant differences between two groups (*P* < 0.05).

Time points are T0 (prior to anesthesia induction); T1 (insertion of the endotracheal tube); T2 (at the commencement of pneumoperitoneum); T3 (10 min after pneumoperitoneum); T4 (30 min after pneumoperitoneum); T5 (at the end of surgery); T6 (removal of the endotracheal tube), and T7 (in PACU).

MBP = mean blood pressure; HR = heart rate. PACU = post-anesthesia care unit.

**Table 6:** Episodes of vital sign instability.

Characteristic	Group C (*n* = 38)	Group E (*n* = 36)	*P-*value
Intraoperative period			
Hypotension, *n* (%)	16 (42.11)	14 (38.89)	0.778
Hypertension, *n* (%)	4 (10.53)	0	0.115
Bradycardia, *n* (%)	4 (10.53)	6 (16.67)	0.510
Tachycardia, *n* (%)	4 (10.53)	2 (5.56)	0.675
Hyoxemia, *n* (%)	0	0	/
Postoperative period			
Hypotension, *n* (%)	0	0	/
Hypertension, *n* (%)	2 (5.26)	0	0.494
Bradycardia, *n* (%)	0	0	/
Tachycardia, *n* (%)	0	0	/
Hyoxemia, *n* (%)	0	0	/

Data are given as *n* (%).

**Figure 5.**
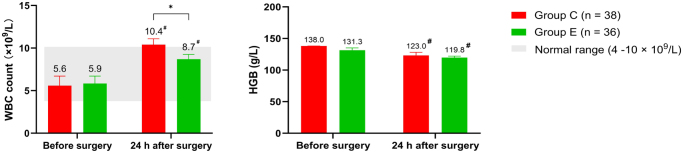
Perioperative blood routine test.

Postoperatively, both groups showed an increase in WBC count and a decrease in HGB levels compared to preoperative values. However, the degree of WBC elevation was significantly lower in Group E than in Group C (Fig. 5).

**#** Significant differences were observed compared to preoperative baseline.

* Significant differences between two groups (*P* < 0.05).

WBC = white blood cell. HGB = hemoglobin.

## Discussion

In this study, the patients managed using the ERAS protocol had a significantly shorter LOS than those in the conventional care group and the 40.26% LOS reduction may meet the minimal clinically important difference (MCID) threshold. Furthermore, implementation of the ERAS protocol was associated with improved quality of recovery, opioid-sparing effects, reduced postoperative complications and inflammatory responses, and diminished postoperative pain.

Traditionally, most patients undergoing LSC have an average postoperative LOS of 1–4 days for safety reasons^[20–22]^. However, with advancements in medical technology and implementation of the ERAS protocol, there has been a recent shift towards outpatient surgeries for POP, particularly in patients undergoing minimally invasive procedures^[23]^. Our study is the first to apply a prospective RCT to LSC, providing greater evidence-based medical value in demonstrating the clinical efficacy of ERAS. Moreover, the positive findings support the feasibility and benefits of the ERAS protocol in LSC, aligning with and expanding upon the current literature^[24–26]^. Notably, the ERAS group achieved a reduction of approximately 31 hours (40.26%) in postoperative LOS relative to the conventional group. While the MCID for LOS has not been formally defined, prior enhanced recovery studies have reported average reductions of about 30–50% as representing meaningful improvements in patient recovery and hospital efficiency^[27]^. The current findings therefore indicate both statistically and clinically significant benefits of ERAS implementation in LSC.

Although the ERAS group had lower hospitalization costs than the conventional group, this difference was not statistically significant. The breakdown of hospitalization costs in both the ERAS and the conventional group revealed that the ERAS protocol, while associated with a 30.5-hour reduction in LOS, resulted in savings in bed fees, diagnostic, and examination costs (approximately 202 RMB). Moreover, ERAS led to savings in postoperative PCIA analgesia costs (224 RMB). In total, the ERAS group saved approximately 426 RMB in hospitalization costs. However, the implementation of multimodal analgesia in the ERAS protocol, which included oral medications and TAP block, incurred additional diagnostic and medication costs of approximately 402 RMB. This increase in costs might be offset by the savings achieved from the reduced LOS, resulting in no significant difference in total hospitalization costs^[28]^. This suggests that while ERAS provides some cost-saving benefits, the additional costs related to multimodal analgesia may neutralize these savings, accounting for the nonsignificant difference between the two groups.

The beneficial effects of rehabilitation evaluated by QoR-15 scores were sustained for up to 7 days postsurgery. Although the improvement was modest, it may reflect better postoperative analgesia and overall comfort. However, there was no significant difference in the QoR-15 scores between the two groups at 30 days postoperatively, suggesting that the ERAS approach may have a limited impact on long-term outcomes in reconstructive pelvic surgery^[24,29]^.

POUR is a common complication of pelvic reconstructive surgery and a significant barrier to early discharge^[30]^. Intraoperative pelvic manipulation and stimulation of the vesicourethral and pelvic nerves can interfere with postoperative spontaneous voiding, whereas anesthetic drugs that affect the autonomic nervous system may result in weak bladder contractions^[31]^. Additionally, the incidence of POUR is higher in POP surgeries than in other pelvic procedures, particularly anterior pelvic reconstructive surgeries. This is likely due to changes in the urethrovesical junction (UVJ) angle, which can cause urethral obstruction^[12]^. In cases of POP surgery without concurrent procedures for incontinence, the incidence of POUR can be as high as 29%^[32]^. Consequently, the duration of urinary catheterization is often longer in these patients than in those undergoing other types of pelvic surgery. Currently, there is no consensus on the optimal duration of urinary catheterization after LSC. Early removal of the urinary catheter, as commonly practiced in the ERAS protocols for other surgeries, is challenging in POP procedures because of the increased risk of POUR. For example, one RCT found that women who had their catheters removed 6 hours after robotic POP surgery were more likely to experience urinary retention and urinary tract infections than those who had their catheters removed on postoperative day one^[33]^. In our study, we shortened the duration of catheter indwelling to 24 h postoperatively in the ERAS group and to >24 h in the conventional group, and no patients required re-catheterization during hospitalization. Moving forward, we may explore reducing the catheterization time to further shorten the LOS.

PONV is a common complication in female patients after laparoscopic surgery, with an incidence of 15–43%^[34,35]^. In our study, fewer patients experienced PONV, which may be attributed to the prophylactic use of two or more antiemetic medications^[36]^, and the opioid-sparing effects of the multimodal analgesia approach employed in the ERAS protocol^[19]^.

Perioperative pain management is a crucial component of the ERAS protocol, and effective analgesia can significantly reduce perioperative stress and enhance patient recovery^[37]^. We used oral NSAIDs as preemptive and postoperative analgesics in the ERAS group, replacing traditional postoperative opioid analgesic pumps. Additionally, we incorporated intravenous lidocaine infusion and TAP blocks to achieve opioid-sparing effects and provide superior pain relief. Lidocaine is known for its anti-inflammatory properties, and its use in multimodal analgesia has been associated with a significant reduction in procedure-induced proinflammatory cytokine release and lower C-reactive protein levels^[38]^. A study by Lin *et al* demonstrated that intraoperative low-dose intravenous lidocaine administration in patients undergoing laparoscopic surgery significantly reduced fentanyl consumption and improved postoperative pain^[39]^. Consistent with our results, another study reported that patients in the lidocaine group had lower WBC counts on the first postoperative day^[40]^. Moreover, TAP blocks have been shown to improve both early and late postoperative pain at rest after minimally invasive surgeries, and this type of pain control can indirectly reduce the incidence of postoperative complications such as delirium, pneumonia, urinary retention, and falls^[41]^. Our findings also confirmed the effectiveness of multimodal analgesia, with this superior analgesic effect extending up to the 7th postoperative day.

This study had several limitations. This single-center prospective trial focused on a specific patient population, which may limit the generalizability of the findings. Multicenter studies are required to validate these results in a broad cohort of patients. As most candidates for LSC are middle-aged or younger adults, our trial included patients aged under 60 years with ASA I–II, limiting generalizability to older or frail populations. The restricted generalizability of our findings should be considered when interpreting the results, particularly regarding ecological validity. This is because POP is most prevalent and surgically managed in older women. A nationwide multicenter survey in China reported that symptomatic POP affects 9.6% of the adult female population, with a notably higher prevalence among women aged ≥60 years^[42]^. Furthermore, the peak incidence for POP surgery is observed in women between 60 and 79 years of age (31 per 10 000 women)^[43]^. Consequently, our study cohort underrepresents this key demographic. For these older or frailer patients, ERAS pathways may need to be modified by incorporating more gradual mobilization schedules, optimized multimodal analgesia, and intensified perioperative monitoring. In addition, due to limited medical resources and a more conservative approach to urinary catheter removal, patients in the ERAS group did not achieve same-day discharge, as reported in other studies^[44]^. Finally, this study only monitored routine blood tests and did not assess perioperative inflammatory markers such as C-reactive protein (CRP) and interleukin-6 (IL-6), which directly reflect the body’s inflammatory response.

## Conclusion

The implementation of a multidisciplinary ERAS protocol in elective LSC demonstrated significant advantages over conventional perioperative management. The ERAS group experienced shorter postoperative LOS and faster recovery as well as a notable reduction in postoperative complications, pain, and inflammatory responses and reduction in opioid use. And the 30-day readmission and ED visit rates remained zero, further confirming the safety of the ERAS protocol.

## Data Availability

The original recorded data will be uploaded on the clinical trial public management platform ResMan Research Manager within 6 months of publication. ResMan Research Manager: http://www.medresman.org.cn/. The de-identified dataset and statistical analysis plan are currently accessible via a provisional repository link for the purpose of peer review:10.6084/m9.figshare.30784145.

## Research registration unique identifying number (UIN)

This study was registered with the Chinese Clinical Trial Registry (Registration number: ChiCTR2400087431, the URL of registration is https://www.chictr.org.cn/showproj.html?proj=228919). Due to the delayed registration, the full protocol, blank case report forms (CRFs), and ethics approval letter are provided as Supplementary Material 1 for editorial review.
